# Socioeconomic Inequalities in Older Adults' Health: The Roles of Neighborhood and Individual-Level Psychosocial and Behavioral Resources

**DOI:** 10.3389/fpubh.2019.00318

**Published:** 2019-10-25

**Authors:** Chun-Qing Zhang, Pak-Kwong Chung, Ru Zhang, Benjamin Schüz

**Affiliations:** ^1^Department of Sport and Physical Education, Faculty of Social Sciences, Hong Kong Baptist University, Kowloon Tong, Hong Kong; ^2^School of Psychology, Faculty of Health Sciences, Curtin University, Bentley, WA, Australia; ^3^Department of Sports Science and Physical Education, Faculty of Education, The Chinese University of Hong Kong, Hong Kong, China; ^4^Institute for Public Health and Nursing, University of Bremen, Bremen, Germany

**Keywords:** health inequalities, healthy aging, elderly health, social disadvantage, social inequalities

## Introduction

Health inequalities in older adults are a critical health issue given the fact that the proportion of older adults is growing fast in virtually every country worldwide (1). Health inequalities can be understood as differences in health between groups defined by social structural variables, such as education, income, or ethnicity (2). Generally, individuals with lower socioeconomic status (SES) experience worse overall health, higher levels of morbidity, and more premature mortality, which are particularly relevant in older adults (3–6). There are two competing approaches to understanding inequality effects on health over the lifespan. The cumulative advantage/disadvantage hypothesis proposes that social disadvantage accumulates over the lifespan, leading to more inequality in a range of health outcomes in older age—depending on which indicator of inequality is examined (5, 6). In contrast, the age-as-leveler hypothesis assumed that socioeconomic differences in health between individuals decrease with older age due to more equity through pensions and healthcare in old age (7, 8). Given that socioeconomic differences in health outcomes are potentially avoidable (9), it is particularly important to examine the roles of socio-economic, socio-psychological, and behavioral resources in alleviating health inequalities among older adults from different SES backgrounds (10).

While the overall picture seems clear—lower socioeconomic status is associated with overall worse health, less is known about the association of specific indicators of SES, and health (4). Here in particular, differential effects of individual-level inequality dimensions such as education or wealth, and inequality dimensions determined by an individual's environment can be expected (5). Individual-level indicators of SES might carry information with regards to individual resources, access, and information processing capabilities. Environmental indicators are related to the built or social environment, such as differences in social cohesion, differences in physical access to and availability of social support and other environmental factors (e.g., street connectivity, mixed land use, and inclusion of green spaces) (11, 12). What is more, environments can change over the course of time, as individuals are for example exposed to different environmental factors at work compared to their home, or on the commute—so called momentary environments (13).

Of particular interest in this context is the existence and shape of interactions between individual and neighborhood-level SES. In general, living in a less disadvantaged neighborhood seems to profit health above and beyond individual measures of inequality (14). That is, lower SES individuals might benefit more from living in higher SES neighborhoods than individuals with higher SES. The idea is that restrictions in access to health-related resources through individual-level disadvantage, such as lack of funds for medical treatments, could be buffered by district-level resources, such as access to a neighborhood-level resource (e.g., community health nurse) (14). Although the influence of neighborhood-level SES on health is smaller than individual-level SES, it has been demonstrated that systematic health inequalities exist between neighborhoods differing in SES (11). Individuals living in more affluent neighborhoods experience better health and lower rates of mortality and morbidity than those living in more disadvantaged areas (11). This highlights that the field requires a more systematic approach toward examining the relationship between SES and health on multiple levels to develop and improve health promotion and disease prevention programs from an early stage (15). However, it is unclear how individual-level SES, neighborhood SES, and their interactions affect older adults' health.

## Psychological, social, and behavioral resources

How do social inequalities translate into health differences? Apart from obvious differences in the access to resources for health (e.g., health care), psychological, social, and behavioral resources seem to be key mediators between socioeconomic inequalities and health.

Older adults' SES might have an impact on their psychological resources such as optimistic self-beliefs, self-efficacy, and perceived autonomy, which can in turn influence their health (16–18). For example, self-efficacy can promote older adults participating in health behaviors as they believe that they are able to take action to control environmental challenges and demands (16). Similarly, older adults who have hope for the future (i.e., optimistic future expectation) tend to report higher levels of health (17). Moreover, older adults' perceived autonomy is also believed to positively affect older adults' health via buffering negative influences of life stressors (18). However, most research in this area has only focused on the mediation between individual-level measures of SES and health via self-efficacy, with very few studies examining whether and how neighborhood socioeconomic characteristics impact on self-efficacy, let alone health (19).

Social resources, such as social support, interpersonal trust, and social cohesion can act as a buffer mechanism of the effects of inequality on health. For example, the availability of instrumental (e.g., providing concrete help such as assistance with travel), informational (e.g., providing relevant information for a health problem), or affective (e.g., consoling in the face of a health problem) social support could provide some of the resources that would otherwise be inaccessible to more disadvantaged individuals (20). This buffering mechanisms of social support also emphasize the role of an environmental perspective—social environments such as neighborhood social cohesion are particularly relevant sources of social support (21). Social cohesion refers to social bonds among individuals in the same neighborhood. It has been demonstrated that both individual and neighborhood-level social cohesion mediate the relationship between neighborhood contexts and individuals' health outcomes (22).

Besides stress-related pathways, health inequalities are also related to socioeconomic differences health behaviors (11). For example, the British Whitehall II longitudinal cohort study demonstrated that health behaviors (i.e., smoking, alcohol consumption, dietary, and physical activity) substantially explained the effects of SES on mortality (23).

Differences in neighborhood resources have consistently been associated with differences in the health of older adults (24). It has been demonstrated that the impacts of neighborhood SES on individuals' health can be translated through his or her psychological and social resources (25), as well as behavioral resources (26). Beyond the direct effects of neighborhood SES on health, it can be assumed that neighborhood-level SES moderate the indirect effects from individual-level SES on the health of older adults via psychological, social, and behavioral resources. For example, systematic difference might exist among older adults who live in high SES neighborhoods to have both higher levels of psychological resources (e.g., self-efficacy and optimistic self-beliefs), higher levels of social resources (e.g., interpersonal trust and social support), and at the same time better access to behavioral resources for promoting their health (e.g., access to healthy food environments and sports facilities).

## A theoretical model on individual and neighborhood SES, psychological, social, and behavior resources, and health

**Figure 1:**
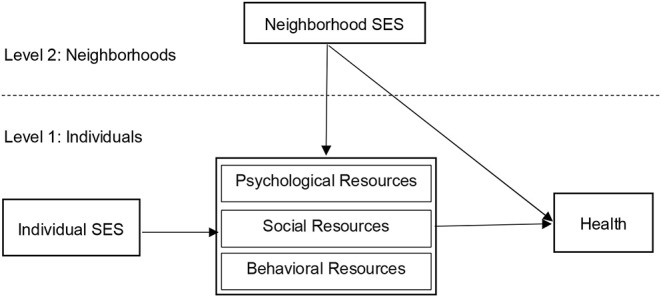
Theoretical model of the relationship between individual and neighborhood SES, psychological, social, and behavioral resources, and health.

Effects of individual-level SES on health (20) and neighborhood-level SES on health (14) have been examined independently, and tests of interactive effects on behavioral resources and health are rare (27). There is also a lack of research examining the contributions of protective psychological, social, and behavioral resources as potentially parallel and/or multiple mediators between social inequality and health in older adults. In this opinion paper, we propose a model (see Figure 1) that outlines the effects of both individual and neighborhood SES on older adults' health as both main effects and interactions.

In this theoretical model, we suggest that psychological, social, and behavioral resources serve as mediators from individual-level SES to health in the proposed model. This is because it has been consistently demonstrated that people from low SES tend to have lower levels of psychological resources (e.g., perceived autonomy), lower levels of social resources (e.g., neighborhood cohesion), and lower levels of behavioral resources (e.g., physical activity) (21–23). We also suggest that these mediating effects will be moderated by neighborhood SES, given the systematic contextual inequalities on health and the determinants (resources) of health (28). Although these multiple levels of SES and their interactions with person-level resources are well-known and well-researched as determinants of health and well-being in environmental gerontology (29), the assumption of mediation and moderated mediation in the model are adapted from previous psychosocial theories. One notable example is social cognitive theory (30) that contributes to our understanding of the cognitive, vicarious, self-reflective, and self-regulatory processes through which social inequality is translated into health and well-being via the human agency.

There are methodological challenges to such designs—unknown sources of errors and further weaknesses in the study design might affect the estimation of the ecological effects within this theoretical model and lead to over- or underestimation of environmental influences (31). In particular, confounding composition and contextual effects are problematic (28). Compositional effects of environmental factors describe effects that are due to the individuals that compose or inhabit in a particular environment—for example, more individuals with higher educational attainment living in a more affluent neighborhood. Health differences between this affluent neighborhood and a more deprived neighborhood might then be due to differences in individual educational attainment. Contextual effects on the other hand are those that are due to proprietary features of the environment, such as better air quality or closer proximity to green spaces. In addition to confounding effects, a lack of variation in ecological exposure, an unbalanced number of ecological units and adults within each unit would as well as multicollinearity of within- and between-indicators would affect estimation of effects (31).

There are threats to the causal claims of multilevel studies of neighborhood influences on health, in particular the endogeneity (32). For example, findings on the relations among key constructs in a model might be distorted due to the endogeneity problem of reverse causality using cross-sectional data (3, 17, 26). The reverse causality might also be caused due to the issue of selection, for example, older adults move to a particular neighborhood because of their existing health status. More often, endogeneity is caused due to the unobserved (confounding) variables that are related to both the neighborhood exposures and health (32). Solutions to overcome the problem of endogeneity in the non-experimental settings are: (a) collecting and controlling a comprehensive range of unobserved variables, (b) using an instrumental variable estimation, (c) using the propensity score matching approach, or better (d) using quasi-experimental designs (32, 33). That said, Antonakis and colleagues recommended 10 best practices for making causal inferences in research in social sciences and the related public health research, and they also suggested that if possible researchers should use the Monte Carlo analysis concurrently (33).

## Future directions and conclusion

We need to know more about the relative impact of individual and contextual indicators of SES in determining health in older adults, and how exactly these SES indicators influence psychosocial mediators of health. These mediators and the circumstances under which they work (e.g., the moderators present in the neighborhood), are crucial in order to devise effective interventions to reduce health inequalities in old age. The proposed model holds an interactive perspective by integrating an individual-level with an environment-level perspective on how social inequalities affect health.

Looking forward, formative research on this integrated model of the effects of individual and neighborhood SES on older adults' health (see Figure 1) can help identify those determinants that hold the most promise as intervention targets, and specify neighborhood resources that might serve to compensate for individual shortcomings in these determinants (34). In addition, special considerations should be given to older adults' savings, pensions, and support from their children and government. This is because many conventional markers of individual and neighborhood-level SES including income, education, and occupation might be different for older adults as compared to the younger populations (24). Moreover, a life course perspective should be considered so that the influence of neighborhood SES on older adults' health is considered not static but as a dynamic process with variations (35). Lastly and importantly, context-sensitive investigations of the effects of individual and neighborhood-level SES on old adults' health is needed in order to inform researchers and policy makers when designing research project for reducing the health inequalities in older adults. For example, a large scale investigation of socioeconomic inequalities in the rates of death and self-assessment of health among 22 European countries indicates that substantial variation in the magnitude of inequalities in SES and health behaviors exist among different countries. Individuals from lower SES countries had substantially higher death rates and poorer self-evaluated health than from countries with higher SES (36).

We hope this opinion paper contributes to the theoretical understanding of the influences of individual and neighborhood SES and their interactions on the health of older adults. Most importantly, we call for more studies examining whether and how the psychological, social, and behavioral resources can serve as changing pathways for the effects from both individual and neighborhood-level SES to older adults' health.

## Author Contributions

C-QZ conceived of this opinion piece and co-wrote the manuscript with BS. P-KC and RZ contributed further ideas and had editorial input into this opinion paper. All authors read and approved the final manuscript and agree with the order that authors are presented.

### Conflict of Interest

The authors declare that the research was conducted in the absence of any commercial or financial relationships that could be construed as a potential conflict of interest.

## References

[1] Bengtson V Global Aging and Challenges to Families. New York, NY: Routledge (2018). 10.4324/9781351328166

[2] Braveman P. Health disparities and health equity: concepts and measurement. Annu Rev Public Health. (2006) 27:167–94. 10.1146/annurev.publhealth.27.021405.102103 16533114

[3] Zimmer Z Kwong J. Socioeconomic status and health among older adults in rural and urban China. J Aging Health. (2004) 16:44–70. 10.1177/0898264303260440 14979310

[4] Acciai F. The age pattern of social inequalities in health at older ages: are common measures of socio-economic status interchangeable? Public health. (2018) 157:135–41. 10.1016/j.puhe.2018.01.002 29524811

[5] Cullati S. Socioeconomic inequalities in health trajectories in Switzerland: are trajectories diverging as people age? Sociol Health Illn. (2015) 37:745–64. 10.1111/1467-9566.12232 25683678

[6] Della Bella S Lucchini M Assi J Health inequality across time: a growth curve analysis of self assessed health in contemporary Switzerland. Swiss J Sociol. (2012) 32:291–309.

[7] Ross CE Wu CL. Education, age, and the cumulative advantage in health. J Health Soc Behav. (1996) 37:104–20. 10.2307/2137234 8820314

[8] Herd P Do functional health inequalities decrease in old age? Educational status and functional decline among the 1931–1941 birth cohort. Res Aging. 2006;28:375–92. 10.1177/0164027505285845

[9] Artazcoz L Rueda S. Social inequalities in health among the elderly: a challenge for public health research. J Epidemiol Community Health. (2007) 61:466–67. 10.1136/jech.2006.058081 17496251 PMC2465705

[10] Grundy E Sloggett A. Health inequalities in the older population: the role of personal capital, social resources and socio-economic circumstances. Soc Sci Med. (2003) 56:935–47. 10.1016/S0277-9536(02)00093-X 12593868

[11] Diez Roux AV Mair C. Neighborhoods and health. Ann N Y Acad Sci. (2010) 1186:125–45. 10.1111/j.1749-6632.2009.05333.x 20201871

[12] Gelormino E Melis G Marietta C Costa G. From built environment to health inequalities: an explanatory framework based on evidence. Prev Med Rep. (2015) 2:737–45. 10.1016/j.pmedr.2015.08.019 26844145 PMC4721462

[13] Saarloos D Kim JE Timmermans H. The built environment and health: introducing individual space-time behavior. Int J Environ Res Public Health. (2009) 6:1724–43. 10.3390/ijerph6061724 19578457 PMC2705214

[14] Boylan JM Robert SA. Neighborhood SES is particularly important to the cardiovascular health of low SES individuals. Soc Sci Med. (2017) 188:60–8. 10.1016/j.socscimed.2017.07.005 28732236 PMC5563460

[15] Braveman PA Egerter SA Cubbin C Marchi KS. An approach to studying social disparities in health and health care. Am J Public Health. (2004) 94:2139–48. 10.2105/AJPH.94.12.2139 15569966 PMC1448604

[16] Grembowski D Patrick D Diehr P Durham M Beresford S Kay E. Self-efficacy and health behavior among older adults. J Health Soc Behav. (1993) 34:89–104. 10.2307/2137237 8277130

[17] Steptoe A Wright C Kunz-Ebrecht SR Iliffe S. Dispositional optimism and health behaviour in community-dwelling older people: associations with healthy ageing. Br J Health Psychol. (2006) 11:71–84. 10.1348/135910705X42850 16480556

[18] Warner LM Ziegelmann JP Schüz B Wurm S Tesch-Römer C Schwarzer R. Maintaining autonomy despite multimorbidity: self-efficacy and the two faces of social support. Eur J Ageing. (2011) 8:3–12. 10.1007/s10433-011-0176-6 28798638 PMC5547307

[19] Boardman J Robert S Neighborhood socioeconomic status and perceptions of self-efficacy. Sociol Perspect. (2000) 43:117–36. 10.2307/1389785

[20] Schöllgen I Huxhold O Schüz B Tesch-Römer C. Resources for health: differential effects of optimistic self-beliefs and social support according to socioeconomic status. Health Psychol. (2011) 30:326–35. 10.1037/a0022514 21553976

[21] Kawachi I. Social capital and community effects on population and individual health. Ann N Y Acad Sci. (1999) 896:120–30. 10.1111/j.1749-6632.1999.tb08110.x 10681893

[22] Rios R Aiken LS Zautra AJ. Neighborhood contexts and the mediating role of neighborhood social cohesion on health and psychological distress among Hispanic and non-Hispanic residents. Ann Behav Med. (2012) 43:50–61. 10.1007/s12160-011-9306-9 22037963

[23] Stringhini S Sabia S Shipley M Brunner E Nabi H Kivimaki M. Association of socioeconomic position with health behaviors and mortality. JAMA. (2010) 303:1159–66. 10.1001/jama.2010.297 20332401 PMC2918905

[24] Yen IH Michael YL Perdue L. Neighborhood environment in studies of health of older adults: a systematic review. Am J Prev Med. (2009) 37:455–63. 10.1016/j.amepre.2009.06.022 19840702 PMC2785463

[25] Robert SA. Neighborhood socioeconomic context and adult health: the mediating role of individual health behaviors and psychosocial factors. Ann N Y Acad Sci. (1999) 896:465–8. 10.1111/j.1749-6632.1999.tb08171.x 10681952

[26] Fisher KJ Li F Michael Y Cleveland M. Neighborhood-level influences on physical activity among older adults: a multilevel analysis. J Aging Phys Act. (2004) 12:45–63. 10.1123/japa.12.1.45 15211020

[27] Schüz B. Socio-economic status and theories of health behaviour: time to upgrade a control variable. Br J Health Psychol. (2017) 22:1–7. 10.1111/bjhp.12205 28059490

[28] Diez Roux AV. Investigating neighborhood and area effects on health. Am J Public Health. (2001) 91:1783–9. 10.2105/AJPH.91.11.1783 11684601 PMC1446876

[29] Wahl HW Iwarsson S Oswald F. Aging well and the environment: toward an integrative model and research agenda for the future. Gerontologist. (2012) 52:306–16. 10.1093/geront/gnr154 22419248

[30] Bandura A. Human agency in social cognitive theory. Am Psychol. (1989) 44:1175–84. 10.1037//0003-066X.44.9.1175 2782727

[31] Blakely TA Woodward AJ. Ecological effects in multi-level studies. J Epidemiol Community Health. (2000) 54:367–74. 10.1136/jech.54.5.367 10814658 PMC1731678

[32] Kawachi I Subramanian SV. Neighbourhood influences on health. J Epidemiol Community Health. (2007) 61:3–4. 10.1136/jech.2005.045203 17183006 PMC2465584

[33] Antonakis J Bendahan S Jacquart P Lalive R On making causal claims: a review and recommendations. Leadersh Q. (2010) 21:1086–120. 10.1016/j.leaqua.2010.10.010

[34] Dehar MA Casswell S Duignan P Formative and process evaluation of health promotion and disease prevention programs. Eva Rev. (1993) 17:204–20. 10.1177/0193841X9301700205

[35] Robert SA Cagney KA Weden MM A life-course approach to the study of neighborhoods and health. In: Bird CE Conrad P Fremont AM Timmermans S, editors. Handbook of Medical Sociology. Nashville: Vanderbilt University Press (2010). p. 124–43.

[36] Mackenbach JP Stirbu I Roskam AJR Schaap MM Menvielle G Leinsalu M. Socioeconomic inequalities in health in 22 European countries. N Engl J Med. (2008) 358:2468–81. 10.1056/NEJMsa0707519 18525043

